# Interactions between genetic admixture, ethnic identity, APOE genotype and dementia prevalence in an admixed Cuban sample; a cross-sectional population survey and nested case-control study

**DOI:** 10.1186/1471-2350-12-43

**Published:** 2011-03-24

**Authors:** Beatriz Marcheco Teruel, Juan J Llibre Rodríguez, Paul McKeigue, Teresa Collazo Mesa T, Evelyn Fuentes, Adolfo Valhuerdi Cepero A, Milagros A Guerra Hernandez, John RM Copeland JRM, Cleusa P Ferri, Martin J Prince

**Affiliations:** 1 National Centre for Medical Genetics, Havana, Cuba; 2 Higher Institute of Medical Sciences of Havana, Cuba; 3 Public Health Sciences section, University of Edinburgh Medical School, Edinburgh, UK; 4 Medical Sciences University of Matanzas, Matanzas, Cuba; 5 University Policlinic "27 de Noviembre", Havana, Cuba; 6 University of Liverpool, Liverpool, UK; 7 King's College, London, (Institute of Psychiatry, Centre for Global Mental Health), UK

## Abstract

**Background:**

The prevalence and incidence of dementia are low in Nigeria, but high among African-Americans. In these populations there is a high frequency of the risk-conferring APOE-e4 allele, but the risk ratio is less than in Europeans. In an admixed population of older Cubans we explored the effects of ethnic identity and genetic admixture on APOE genotype, its association with dementia, and dementia prevalence.

**Methods:**

A cross-sectional catchment area survey of 2928 residents aged 65 and over, with a nested case-control study of individual admixture. Dementia diagnosis was established using 10/66 Dementia and DSM-IV criteria. APOE genotype was determined in 2520 participants, and genetic admixture in 235 dementia cases and 349 controls.

**Results:**

Mean African admixture proportions were 5.8% for 'white', 28.6% for 'mixed' and 49.6% for 'black' ethnic identities. All three groups were substantially admixed with considerable overlap. African admixture was linearly related to number of APOE-e4 alleles. One or more APOE-e4 alleles was associated with dementia in 'white' and 'black' but not 'mixed' groups but neither this, nor the interaction between APOE-e4 and African admixture (PR 0.52, 95% CI 0.13-2.08) were statistically significant. Neither ethnic identity nor African admixture was associated with dementia prevalence when assessed separately. However, considering their joint effects African versus European admixture was independently associated with a higher prevalence, and 'mixed' or 'black' identity with a lower prevalence of dementia.

**Conclusions:**

APOE genotype is strongly associated with ancestry. Larger studies are needed to confirm whether the concentration of the high-risk allele in those with African ancestry is offset by an attenuation of its effect. Counter to our hypothesis, African admixture may be associated with higher risk of dementia. Although strongly correlated, effects of admixture and ethnic identity should be distinguished when assessing genetic and environmental contributions to disease risk in mixed ancestry populations.

## Background

In the only detailed population-based studies from sub-Saharan Africa, the prevalence and incidence of Alzheimer's Disease (AD) and dementia in Nigeria are very low [1,2]. However, among African-Americans, prevalence and incidence rates are similar to [1,2], or even higher [3,4] than the rates for white non-Hispanic Americans. Also, the prevalence of dementia in some Caribbean and South American populations with African admixture, are among the highest in the world [5-8].

The e4 allele of the apolipoprotein-E gene was first reported to be associated with an increased risk of AD twenty years ago [9,10]. Since then, this has been the most consistently replicated genetic risk factor [11]. The association has been observed in many different populations [12]. However, in African-Americans, other populations of west African ancestry, and Hispanics the association of AD with e4 is relatively weak and inconsistent, even though the frequency of the risk-conferring APOE e4 allele is higher in those of African ancestry than in other continental groups [13]. No association of APOE genotype with AD has been observed in studies in Nigeria [14], or Kenya [15]. Two longitudinal studies in the US also suggest no association between APOE e4 and incident AD among African Americans, while the incidence of AD seemed to be higher for African Americans in every APOE genotype [3,4]. In a case-control study in Florida the association between APOE e4 and AD was as strong for Cuban Americans as for white non-Hispanics [16] in contrast to the absence of an observed association among Hispanics in North Manhattan [4]. This pattern of findings is strongly suggestive of the presence of gene by environment, and/or gene by gene interactions [17].

Those classified in the US as 'Hispanic' originate from diverse mixed ancestry Caribbean, Central and South American populations, resulting from two-way admixture between Native American and European populations or three-way admixture among Native American, European, and West African populations [18]. However, patterns of admixture vary greatly among these populations. The catch-all 'Hispanic' category is therefore problematic, providing some information about linguistic and cultural heritage but very little about ancestry. In much of continental Latin America, two-way admixture dominates with little evidence of African ancestry [19]. Cuba is quite different. The first European contact was by Columbus in 1492. The indigenous population was reputedly extinct by 1700. While the ancestral Native American substrate is still appreciable in the maternal lineages, the extensive process of population admixture in Cuba has left no trace of paternal Native American lineages, mirroring the strong sexual bias in the admixture processes taking place during colonial times [20]; currently Native American admixture is minimal [21]. Importation of slaves from West Africa was current by 1600 and not abolished until 1886. The proportion of population identified as black or mixed rose from 34% in 1774 to 57% by 1817. Recent Cuban studies concur in identifying average proportions of African admixture in those who classify themselves as white, mixed race and black as, respectively, about 5%, 35% and 60% [20,21]. European admixture among African-Americans is much lower, an average of between 12% and 20% in different US cities [22] and very few African-Americans have as much as 50% European ancestry [23]. In the former British Caribbean, average European admixture levels may be even lower; just 7% in Jamaica [22].

The high levels of African and European admixture in Cuba can be used to good effect. Studying the relationship of dementia risk to individual admixture within admixed populations is the most direct way to distinguish genetic from environmental explanations for ethnic differences in disease risk [24], and, by extension, for distinguishing gene by environment versus gene by gene explanations for ethnic differences in the effects of genes on disease outcomes. Furthermore, such relationships will confound studies of other genetic risk factors - "hidden population stratification". Measurement of the confounder (individual admixture) allows us to control for population stratification using standard methods.

We recently reported a high prevalence of dementia in a one phase prevalence study in an older Cuban urban population [25]. In the present study we aimed to

1. analyse the association between ethnic group and individual admixture

2. assess the association of each with APOE genotype

3. test the hypotheses that

a. the effect of APOE genotype on dementia is modified by ethnic group, and/or admixture, with weaker associations among those with 'mixed' and 'black' ethnic identity and with higher proportions of African admixture

b. the prevalence of dementia is lower among those with 'mixed' and 'black' ethnic identity, and is inversely linearly related to African admixture

## Methods

### Setting and Study design

A one phase cross-sectional catchment area survey of all those aged 65 years and over living in five catchment areas in Ciudad Havana, Cuba (Lisa, Luyano, Marianao, Playa, and Plaza); and one catchment area in Matanzas (Milanes), a city 120 kilometres east from Havana, and a nested case-control comparison of admixture. This involved estimating individual admixture on all dementia cases and a randomly selected sample of controls free of dementia. This is an efficient design, in that it reduces the cost of the genotyping work while retaining much of the power for the tests of the main hypotheses. All participants received a full assessment lasting approximately 2-3 hours, including a participant interview, a physical examination, phlebotomy, and an informant interview. Interviews were carried out by polyclinic doctors (psychiatrists, geriatricians or general medical specialists) working in the areas selected. Participants were recruited on the basis of informed signed consent. The Medical University of Havana, the National Centre of Medical Genetics (Cuba), and the Institute of Psychiatry institutional review boards reviewed and approved this project. Full details of the protocol for the 10/66 population-based cross-sectional surveys are provided in an open-access online journal publication [26].

### Measures

The 10/66 interview generates information on dementia diagnosis, mental disorders, physical health, anthropometry, demographics, dementia and non-communicable disease risk factors, disability and functioning, health service utilisation, care arrangements and caregiver strain [26]. Only those assessments relevant to the current analysis of dementia, ethnicity, admixture and APOE genotype are described in detail here.

1) Outcome - The diagnosis of dementia.

Dementia was diagnosed according to our own cross-culturally validated 10/66 dementia diagnosis algorithm [27], and according to DSM-IV criteria [28]. A concurrent validation conducted in the course of the Cuban population-based study showed that DSM-IV dementia diagnosis was specific but insensitive to mild to moderate dementia; the 10/66 Dementia diagnosis corresponded better to local clinician diagnosis and was more sensitive to these milder cases [29]. The outcome for most of the analyses in this paper is 'any dementia diagnosis' comprising all those meeting either or both of these criteria. Diagnoses were established following.

(i) A structured clinical interview, the Geriatric Mental State, which applies a computer algorithm (AGECAT)[30], identifying organicity (probable dementia), depression, anxiety and psychosis and,

(ii) A cognitive test battery comprising a) the Community Screening Instrument for Dementia (CSI'D') COGSCORE [31] (incorporating the CERAD animal naming verbal fluency task), and b) the modified CERAD 10 word list learning task with delayed recall [32] and

(iii) An informant interview, the CSI'D' RELSCORE [31] for evidence of cognitive and functional decline, with additional information on dementia onset and course obtained from the modified (Dementia Diagnosis and Subtype) History and Aetiology Schedule [33].

Participants were allocated to the category of 10/66 dementia when they scored above a cutpoint of predicted probability of dementia (>0.25) estimated from the logistic regression equation developed and validated cross-culturally in the 10/66 international pilot study, using coefficients from the GMS, CSI-D informant and cognitive test interviews and the modified CERAD 10 word list learning tasks [27]. DSM-IV dementia is a criterion-based diagnosis requiring impairment in memory and at least one other domain of cognitive function, linked to social or occupational impairment, not better accounted for by delirium or other mental disorder. DSM-IV dementia criteria were applied directly using a computerized algorithm; full details are available in an open access publication [29].

2. Ethnic identity: Participants were classified according to interviewer's perception of ethnic identity, using well-established groups used in the Cuban census - 'Blanco - white', 'Mestizo - mixed' and 'Negro - black'.

3. APOE Genotyping: We aimed to collect 10 ml blood samples from all participants, from which DNA was extracted, quantitated, and archived at the National Centre for Medical Genetics in Havana. Apolipoprotein E genotype was determined using Hhal digestion of amplified products. Genotypes were determined masked to knowledge of clinical phenotypes.

4. Admixture estimation: In a population formed by admixture between two or more founding populations, ancestry informative marker genotype data can be used to estimate the admixture of each individual (the proportion of that individual's genome that has ancestry from each founding population). We aimed to estimate admixture in 600 participants, comprising all dementia cases, and a randomly selected sample of controls. Sixty SNPs were used to estimate individual admixture, chosen from the panel assembled by Dr Mark Shriver at Penn State and Mike Smith at NCI [34]. With an average 40% information content for ancestry, these 60 SNPs would be sufficient to estimate three-way individual admixture proportions with a standard error of less than 0.1 (see additional file 1 for details of individual SNPs and their locations). All genotyping was performed by KBiosciences (Mapple Park, Herts, UK; http://kbioscience.co.uk). SNPs were genotyped using the KASPar chemistry, which is a competitive allele specific PCR SNP genotyping system using FRET quencher cassette oligos (http://www.kbioscience.co.uk/reagents/KASP.html). Plate-identifying blanks and Hardy-Weinberg equilibrium tests were used as quality control tests. The ADMIXMAP program [35] (http://homepages.ed.ac.uk/pmckeigu/admixmap/) was used to generate posterior means of individual admixture from the ancestry informative marker data. In large samples these posterior means are asymptotically equivalent to maximum likelihood estimates.

### Analysis

We describe sample characteristics, comparing those in full survey sample who did and did not provide a blood sample, and for the case-control sub-sample the sampling weights (the inverse of the probability of selection) for dementia cases and controls within each APOE genotype. We describe the proportions assigned to each ethnic identity ('white', 'mixed' and 'black'), and the weighted mean individual admixture proportions (European, African and Native American), and, in the sub-sample, test for an association between them using a weighted one way ANOVA.

We next tested for an association between ethnic identity and APOE genotype and allele frequencies using a Chi-squared test for trend, and an association between APOE genotype and admixture by making a weighted comparison of mean admixture across groups with no, one or two APOE e4 alleles.

We tested for an association between APOE genotype and any dementia with Chi-squared tests and crude and adjusted prevalence ratios derived from a Poisson working model (adjusted for age, sex and educational level). To control for population stratification, the adjusted prevalence ratio for any APOE e4 allele was further adjusted for ethnic identity, and, in the weighted Poisson model in the case-control sub-sample, for admixture. We next estimated the stratum-specific prevalence ratios for the association between any APOE e4 allele and any dementia in the three ethnic identity groups, and fitted a ethnic identity by APOE interaction term to the model. We also fitted an African admixture by APOE interaction term to the weighted Poisson model in the case-control sub-sample.

Finally, we assessed the separate and joint effects of ethnic identity and admixture on dementia prevalence. In the full sample, we describe the crude prevalence of dementia by ethnic identity, and the prevalence of dementia standardised for age, sex and education, and age, sex, education and APOE genotype. Alongside the crude and adjusted prevalence, we also provide prevalence ratios from the analogous Poisson model. In the case-control sub-sample in a weighted analysis we estimated the main effect of admixture (100% African versus 100% European) on dementia controlling for age, sex, education and APOE genotype. We accomplished this by entering both African and North American proportionate admixture into the models, as continuous scales ranging from 0.0 to 1.0, but omitting European admixture. Since proportions for these three variables sum to 1.0 for each participant, European admixture becomes in effect the contrast (akin to the omitted category when using dummy variables), and the coefficient for African admixture is then interpreted as the change in the log prevalence ratio per one unit change in African admixture, that is between 100% African and 100% European admixture. However, the underlying assumption is one of linear variation across this range. Then, in a series of models, we estimated the separate and joint effects of ethnic identity and admixture controlling only for APOE genotype, and then the joint effects controlling also for age, sex and education.

All analysis were carried out using STATA version 9.2.

## Results

### Sample characteristics

Of the 2928 participants completing the survey (an overall response rate of 96.4% of all those eligible), 2520 (86.1%) provided blood samples for APOE genotyping and biomarker analysis. Men (p = 0.07) were slightly under-represented among those not providing blood samples (Table 1). Otherwise, there were no large or statistically significant differences between the two groups regarding education, prevalence of dementia, family history of dementia and prevalence of self-reported stroke, diabetes, hypertension and smoking. Of the 273 people with dementia that provided blood samples 235 were found to be suitable for SNP genotyping, and 349 control samples were selected at random from all non-cases. Of these 235 people with dementia, 231 met criteria for 10/66 dementia, 137 met DSM-IV dementia, and 133 met both criteria. To ensure that estimates would be generalisable, for all subsequent case-control analyses, sample weights were used to weight back for the probability of selection within case and control groups by APOE genotype. For the cases these were APOE e2/e3 1.00; e2/e4 1.00; e3/e3 1.11; e3/e4 1.68; e4/e4 1.14. For the controls these were APOE e2/e3 5.10; e2/e4 3.50; e3/e3 8.16; e3/e4 4.54; e4/e4 2.15. This sampling variation was based on observed frequencies rather than design, and must, presumably, have arisen through chance.

**Table 1:** Sample characteristics, by availability of blood sample

Variables	Without blood sample N = 408	With blood sample N = 2520	P-value (T-test with F-value, or chi-squared test)
Age in years (mean/sd) Missing values	75.4 (7.2) 0	75.0 (7.0) 7	F = 2.4, p = 0.27

Male sex (n/%)	159 (39.0%)	866 (34.4%)	X^2 ^= 3.3, 1 df, p = 0.07

Ethnic identity			X^2 ^= 1.9, 2 df, p = 0.39
'White'	77 (69.4%)	1674 (71.9%)	
'Mixed'	17 (15.3%)	260 (11.2%)	
'Black'	17 (15.3%)	395 (17.0%)	
Missing values	97	191	

Dementia (n/%)	47 (11.5%)	273 (10.8%)	X^2 ^= 0.2, 1 df, p = 0.68

ICD depressive episode (n/%)	19 (4.7%)	125 (5.0%)	X^2 ^= 0.1, 1 df, p = 0.79

Stroke (n/%)	35 (8.6%)	194 (7.7%)	X^2 ^= 0.4, 1 df, p = 0.53
Missing values	2	7	

Diabetes (n/%)	71 (17.5%)	471 (18.8%)	X^2 ^= 0.4, 1 df, p = 0.53
Missing values	2	14	

Hypertension (n/%)	295 (72.3%)	1841 (73.1%)	X^2 ^= 0.1, 1 df, p = 0.75

Smoking (n/%)	176 (43.2%)	1141 (45.4%)	X^2 ^= 0.7, 1 df, p = 0.41
Missing values	1	8	

Family history of dementia (n/%)	75 (18.5%)	473 (18.8%)	X^2 ^= 0.0, 1 df, p = 0.86
Missing values	2	8	

Df = degrees of freedom

### The association between ethnic identity and admixture

**Figure 1:**
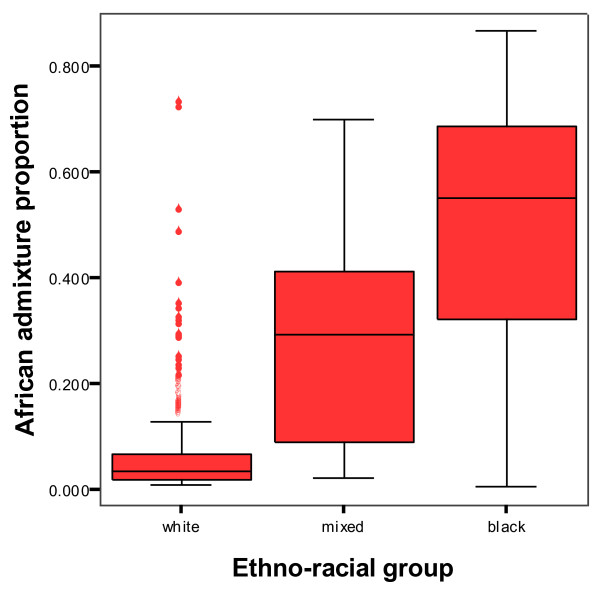
Box plot of African admixture distribution by ethnic identity (weighted)

According to interviewer perceptions, 1677 (72%) were considered to be 'white', while 394 (17%) were considered 'mixed' and 261 (11%) 'black'. For the case-control sub-sample (n = 584), the mean individual admixture proportions (after weighting back) were European 81.2% (95% confidence intervals 79.1-83.3%), African 16.2% (14.1-18.3%), and Native American 2.6% (2.3-3.0%). The mean African admixture proportion for the three ethnic groups was 5.8% (5.1-6.6%) for 'white', 28.6% (24.0-33.2%) for 'mixed' and 49.6% (44.8-55.4%) for 'black'. However, all three groups were substantially admixed with considerable overlap between the three ethnic identities, pure African or European ancestry being the exception (see Figure 1). A small proportion of those defined as 'white' had marked African ancestry and most of those defined as 'black' had inherited much of their genome from European ancestors.

### The association between ethnic identity, admixture and APOE genotype

Table 2 shows the distribution of APOE genotype and APOE allele frequency according to ethnic identity. There was a strong graded association between ethnic identity and APOE genotype, with lower e3 frequency and higher e2 and e4 frequencies moving from 'white' to 'mixed' to 'black' groups. In the case-control sub-sample, there were also graded associations (after weighting back) between individual admixture and APOE genotype. Mean African admixture increased from 0.15 among those with no e4 alleles to 0.19 with one and 0.35 for those with two e4 alleles (Test for trend, F = 4.6, p = 0.01), while mean European admixture declined across the three groups from 0.82 to 0.78 to 0.62 (Test for trend, F = 5.0, p = 0.007). Native American admixture (0.03) did not vary by APOE genotype.

**Table 2:** Distribution of sociodemographic characteristics, APOE genotype and APOE allele frequency by ethnic identity

Ethnic identity	'White' N = 1674	'Mixed' N = 260	'Black' N = 395	**P value X**^**2**^
Age (mean/SD)	75.0 (7.0)	75.7 (7.7)	74.7 (6.9)	F = 1.7, P = 0.19

Male sex (n/%)	579 (34.6%)	95 (36.5%)	118 (29.9%)	*X^2 ^= *4.0, 2df, P = 0.14

Education level (n/%)				*X^2 ^= *25.6, 1 df, P < 0.001
None	37 (2.2%)	7 (2.7%)	14 (3.6%)	
Some	344 (20.6%)	55 (21.2%)	112 (28.5%)	
Primary	543 (32.5%)	87 (33.5%)	148 (37.7%)	
Secondary	429 (25.7%)	62 (23.8%)	73 (18.6%)	
Tertiary	317 (19.0%)	49 (18.8%)	46 (11.7%)	

APOE Genotype (n/%)				*X^2 ^= *31.4, 1 df, P < 0.001
No E4 allele	1423 (85.0%)	222 (85.4%)	298 (75.4%)	
Heterozygous (One e4 allele)	241 (14.4%)	32 (12.3%)	77 (19.5%)	
Homozygous (Two e4 alleles)	10 (0.6%)	6 (2.3%)	20 (5.1%)	

APOE allele frequency				X^2 ^= 42.6, 4 df, P = < 0.001
E2	0.058	0.063	0.072	
E3	0.864	0.852	0.780	
E4	0.078	0.085	0.148	

### The association between APOE genotype and dementia; interactions with ethnic group and admixture

The distribution of APOE genotype and the APOE allele frequency, by dementia status, is shown in table 3. The e2 allele was under-represented, and the e4 allele over-represented among those with dementia. We examined the effect of APOE genotype on dementia prevalence using APOE e3/e3 genotypes as the reference category. After adjusting for age, sex and education, APOE e3/e4 (PR = 2.59, 95%CI 2.04-3.28) and APOE e4/e4 genotypes (PR 2.88, 95% CI1.58-5.27) were strongly associated with dementia. However, there was no apparent protective effect of the e2 allele. The prevalence of dementia was more than double in APOE carriers compared to that in non-carriers (adjusted PR = 2.58, 95%CI 2.06-3.22). This adjusted prevalence ratio was little changed after adjusting also for ethnic identity (PR 2.47, 95% CI 1.96 to 3.12). After weighting back, the association between any APOE e4 allele and dementia, adjusted for age, sex and educational level was naturally similar in the case-control sub-sample, although estimated with less precision (PR 2.54, 95% CI 1.85-3.47). This association was also essentially unchanged after further adjusting for individual admixture (PR 2.57, 95% CI 1.89-3.49).

**Table 3:** APOE Genotype and APOE allele frequency by dementia status, with crude and adjusted prevalence ratios and 95% confidence intervals

		Dementia N(%) 273	No dementia N(%) 2247	Whole sample N(%) 2520	Crude PR (95%CI)	**Adjusted**^**1 **^**PR ** (95%CI)
APOE genotype	e2/e3	24 (8.8%)	255 (11.4%)	279 (11.1%)	0.97 (0.64-1.46)	0.96 (0.64-1.44)
	e2/e4	2 (0.7%)	15 (0.7%)	17 (0.7%)	1.33 (0.36-4.91)	1.42 (0.37-5.45)
	e3/e3	162 (59.3%)	1663 (74.0%)	1825 (72.4%)	1.00 (ref.)	1.00 (ref.)
	e3/e4	77 (28.2%)	285 (12.7%)	362 (14.4%)	2.40 (1.88-3.06)	2.59 (2.04-3.28)
	e4/e4	8 (2.9%)	29 (1.3%)	37 (1.5%)	2.44 (1.30-4.57)	2.88 (1.58-5.27)
	p-value (Test for heterogeneity)				X^2 ^= 54.6, 4 df p < 0.001	X^2 ^= 46.6, 4 df p < 0.001
Number of APOE e4 alleles	0	186 (68.1%)	1918 (85.4%)	2104 (83.5%)	1.00 (ref.)	1.00 (ref.)
	1	79 (28.9%)	300 (13.4%)	379 (15.0%)	2.36 (1.86-2.99)	2.55 (2.02-3.21)
	2	8 (2.9%)	29 (1.3%)	37 (1.5%)	2.45 (1.31-4.58)	2.90 (1.59-5.29)
	p-value (Test for trend)				X^2 ^= 55.5, 1 df p < 0.001	X^2 ^= 41.8, 1 df p < 0.001
Any APOE e4 allele	1 or 2 alleles	87 (31.9%)	329 (14.6%)	416 (16.5%)	2.37 (1.88-2.98)	2.58 (2.06-3.22)
	p-value				X^2 ^= 53.0, 1 df p < 0.001	X^2 ^= 45.6, 1 df p < 0.001
APOE allele frequency	e2	0.048	0.060	0.059		
	e3	0.778	0.860	0.851		
	e4	0.174	0.080	0.090		

	p-value (Test for trend)			X^2 ^= 61.7, 1 df, P < 0.001		

1 Adjusted for age, sex and education - X^2 ^derived from likelihood ratio tests

Stratifying by ethnic identity in the full sample, the association between any APOE-e4 and dementia was similar in 'white' (PR 2.83, 95% CI 2.18-3.68) and 'black' participants (PR 2.38, 95% CI 1.43-3.95), with no association apparent among those rated as having 'mixed' race (PR 0.87, 95% CI 0.25-2.98). However, the likelihood ratio test for the interaction term was not statistically significant (X^2 ^= 4.42, degrees of freedom = 2, p = 0.11). In a sensitivity analysis, after merging the 'mixed' and 'black' groups, the interaction between APOE e4 and ethnic identity showed a non-significant trend towards a weaker APOEe4 dementia association among non-whites compared with whites (PR 0.74, 95% CI 0.45-1.24). In the case-control subsample, extending the model to include an APOE by African ancestry interaction term suggested that the effect (prevalence ratio) of any APOE e4 allele would vary continuously from PR 2.93 (95% CI 1.99-4.31) in those with 100% European ancestry to 1.52 in those with pure African ancestry. However, the interaction term again failed to reach statistical significance (PR 0.52, 95% CI 0.13-2.08), p = 0.36.

### Effects of ethnic identity and admixture on dementia prevalence

There were no statistically significant effects of ethnic identity on dementia prevalence, either before or after adjusting for compositional differences (Table 4). The prevalence of dementia was slightly higher among 'black' participants and slightly lower among 'mixed' participants when compared with 'white participants, these tendencies being amplified after standardizing or adjusting for age, sex and education (Table 4). After further standardizing or adjusting for the compositional differences in APOE genotype, dementia prevalence among both 'black' and 'mixed' groups was slightly lower than among those identified as 'white'. In the case-control subsample the prevalence ratio for 100% African versus 100% European ancestry was PR 0.81 (95% CI 0.41-0.63). After fitting the APOE x African admixture interaction term, the effect of African ancestry was estimated as PR 1.01 (95% CI 0.43-2.39) in those without an APOE e4 allele and 0.52 in those with an APOE e4 allele - albeit that as noted before the interaction term was not statistically significant.

**Table 4:** Crude and adjusted dementia prevalence by ethnic identity, with prevalence ratios

		'White' N = 1677	'Mixed' N = 394	'Black' N = 261	Test for heterogeneity across ethnic identity groups
Crude prevalence					
	Any dementia - crude prevalence (%)	190/1751 10.9% (9.4-12.3%)	25/277 9.0% (5.5-12.5%)	49/412 11.9% (8.8-15.0%)	
	Crude prevalence ratio	1 (ref)	0.83 (0.55-1.25)	1.10 (0.82-1.47)	X^2 ^= 1.0, 2 df p = 0.61
Standardized/adjusted for age, sex and education^1^					
	Any dementia - standardized prevalence (%)	11.1% (9.7-12.5%)	7.8% (5.0-10.6%)	11.8% (8.6-15.1%)	
	Adjusted prevalence ratio	1 (ref)	0.73 (0.50-1.06)	1.04 (0.79-1.38)	X^2 ^= 2.5, 2 df p < 0.29
Standardized/adjusted for age, sex, education and APOE genotype^1^					
	Any dementia - standardized prevalence (%)	11.4% (10.0-12.8%)	8.3% (5.4-11.2%)	9.7% (7.3-12.1%)	
	Adjusted prevalence ratio	1 (ref)	0.74 (0.50-1.10)	0.92 (0.69-1.23)	X^2 ^= 2.2, 2 df p = 0.32

1. X^2 ^derived from likelihood ratio tests

When estimating simultaneously the independent effects of ethnic identity and admixture (controlling for APOE genotype) African admixture was positively associated with dementia prevalence (PR 4.62, 95% CI 1.48-14.5), while estimated prevalence in 'mixed' (PR 0.54, 95% CI 0.30-0.96) and 'black' participants (PR 0.50, 95% CI 0.25-1.00) was significantly lower than among those identified as 'white' (Table 5). The effect of African admixture was slightly attenuated after adjusting for age, sex and education.

**Table 5:** The independent effects of admixture and ethnic identity upon dementia prevalence (weighted analysis)

	Model 1	Model 2	Model 3	Model 4
APOE genotype				

One or more e4 allele	2.21 (1.58-3.09) p < 0.001^1^	2.19 (1.54-3.10) p < 0.001^1^	2.13 (1.50-3.02) p < 0.001^1^	2.45 (1.77-3.40) p < 0.001^1^

Admixture				

100% African versus 100% European admixture	1.53 (0.80-2.91) p = 0.20^1^	-	4.62 (1.48-14.5) p = 0.01^1^	2.55 (0.75-8.61) p = 0.13^1^

Ethnic identity				
'White'	-	1 (ref)	1 (ref)	1 (ref)
'Mixed'	-	0.79 (0.47-1.33) p = 0.38^1^	0.54 (0.30-0.96) p = 0.04^1^	0.60 (0.34-1.09) p = 0.09^1^
'Black'	-	1.02 (0.68-1.51) p = 0.93^1^	0.50 (0.25-1.00) p = 0.05^1^	0.47 (0.22-1.02) p = 0.06^1^

Sociodemographic factors				

Age (years)	-	-	-	1.10 (1.07-1.12) p < 0.001^1^

Male sex	-	-	-	0.84 (0.59-1.21) p = 0.36

Education (per level)	-	-	-	0.76 (0.64-0.90) p = 0.001

1. p-values are taken from the model z-scores. It was not possible to run likelihood ratio tests because robust variance estimates were required given the use of sampling weights

Model 1 African admixture and APOE

Model 2 Ethnic identity and APOE

Model 3 African admixture, ethnic identity and APOE

Model 3 African admixture, ethnic identity, APOE, age, sex and education

## Discussion

There has been much interest in the potential role of African ancestry in modifying the effect of the APOE genotype and influencing risk for AD and other dementias. We believe this to be the first study to have addressed this issue directly, through estimation of individual admixture, rather than relying merely on observations of ethnic type. Another strength is the population-based survey design with high response rates for the main survey and blood sample collection. The main weaknesses of the study were, first, that the sample size, although large, may not have been adequate to exclude important APOE genotype by admixture interaction effects, and second that in the cross-sectional design we were unable to distinguish environmental factors associated with the incidence of dementia from those predicting its duration. Finally, although the use of 60 SNPs to estimate individual admixture is considered to provide reasonable precision, a standard error of around 0.1 is to be expected. Ideally we should have accounted for the uncertainty in these estimates within the regression analysis, rather than treating it as a covariate observed without error. We do not believe that this will have led to systematic error, since most SNPs were successfully genotyped on most participants. Nevertheless, although much more computationally demanding, future more definitive tests of these hypotheses on larger samples would benefit from this more rigorous approach

In this representative population-based survey of older Cubans in Havana and Matanzas cities, ''white', mixed' and 'black' ethnic groups were all substantially admixed, with varying proportions of African and European ancestry. There was a strong and statistically significant association between both ethnic identity and admixture and the APOE genotype, the e4 allele being over-represented in 'mixed' and 'black' ethnic groups and in those with greater African admixture. Overall, we found a strong association between APOE genotype and dementia, with effect sizes very similar to those reported in other settings [11,13]. The association was evident among those identified by interviewers as 'black' as well as those identified as 'white', but not in those identified as 'mixed'. The 'mixed' group was the smallest in number, and lack of precision may have contributed to this otherwise surprising finding. There was a non-significant trend for the association between APOE genotype and dementia to be weaker in those with greater degrees of African admixture. Controlling for ethnic identity or admixture did not affect the association between APOE genotype and dementia, suggesting an absence of confounding by population stratification. After controlling for compositional differences in APOE genotype (the risk conferring e4 allele being more common in 'mixed' and 'black' ethnic identity groups), there was a non-significant trend towards lower dementia prevalence in those 'non-white' groups. A similar non-significant trend was apparent for admixture. However, when the joint independent effects of ethnic identity and admixture were assessed in a single model, mutual confounding was evident. In each ethnic identity, increased African ancestry greatly increased the risk of dementia. At every level of African ancestry, those with 'mixed' and 'black' ethnic identities had a lower risk of dementia

We have established a link between admixture and APOE genotype, with a higher frequency of the risk-conferring e4 allele in those with greater degrees of African admixture. All things being equal, this would be expected to result in a greater incidence and prevalence of dementia. However, in our sample this was offset by a large attenuation of the effect of APOE e4 in those with more African ancestry. This interaction was not statistically significant, and larger samples will be required to measure this with more precision and exclude type II error. Also, there was no significant graded effect modification by ethnic identity in the larger sample, with the attenuation of effect being confined to those in the 'mixed' group. Gene by environment interactions still seem the most plausible explanation for apparent modification of the effect of APOE among African, African American and European populations, given the large difference in environmental exposures, particularly cardiovascular risk factors, between African and African American populations [36]. However, European admixture among African American populations may have also created potential for differential gene by gene interactions between the two settings. Were a more consistent and unequivocal interaction with admixture to be demonstrated in larger samples, we could then in principle begin to localize the genes responsible by admixture mapping, exploiting information about linkage generated by admixture [35].

Another balancing effect on overall prevalence may be in operation given that the effects of 'mixed' or 'black' ethnic identity on the one hand, and African genetic admixture on the other seem to be operating in opposing directions in influencing dementia risk. These are related yet by no means collinear constructs; hence mutual confounding is feasible. The new respectability of observer assessments of 'ethno-race' in epidemiological research arise precisely from their ability to identify the externally observable physical characteristics that are hypothesised to lead to social, economic and health disadvantage. Their utility in research, as well as their limitations are neatly summarised by an American epidemiologist, Camara Phyllis Jones [37]:

'The race that we measure in our studies is the same race that is noted by a taxi driver, a police officer, a judge in a courtroom, or a teacher in a classroom. That is, race is a social classification in our race-conscious society that conditions most aspects of our daily-life experiences and results in profound differences in life chances. This assigned race varies among countries. For example in the United States I am clearly labelled Black, while in Brazil I would be just as clearly labelled White and in South Africa, I would be clearly labelled 'coloured'. It is likely, if I stayed long enough in one of these settings, my health profile would become that of the group to which I had been assigned, even though I would have the same genetic endowment in all three settings.'

Much research in the US has focussed upon black ethnic identity as a socially determined, contextually bound construct, linked to disadvantage and discrimination, and mediating health disadvantages. Thus, in the 1990s darker skin colour among African-Americans was found to be inversely associated with income, education and occupational status, and to be a stronger predictor of adult occupational status than was parental socioeconomic position [38]. More recently, darker skin colour has been shown to be independently associated with experiences of racism [39]. Protective income gradients in hypertension are evident for light-skinned but not dark-skinned African-Americans, an effect hypothesised to be explained by psychosocial stressors linked to skin colour, including racism [40]. Of relevance to our finding of a protective effect of non-white ethnic identity on dementia risk, some benefits of such self-identification have been reported; for example factors reflecting participation in and belonging to African-American culture were associated with a range of positive health behaviours [41].

## Conclusions

Genetic admixture, externally observable physical characteristics including skin colour, and self-reported ethnicity are related to each other, but in complex ways [42,43]. One of the weaknesses in the current study is that we did not adequately separate out self-perceptions from observer ratings of ethnic identity. A proper understanding of the role of genetic admixture in determining disease risk may require measurement of, and control for each of these and other related socio-cultural factors, including socioeconomic position and acculturation [37,44]. Although strongly correlated, the effects of admixture and ethnic identity on health outcomes can and should be distinguished when assessing genetic and environmental contributions.

## Competing interests

The authors declare that they have no competing interests.

## Authors' contributions

TBM, PMcK, JLR and MJP designed the study. TBM carried out the molecular genetic studies. PMcK generated the individual admixture estimates and supported the admixture analyses. JLB and AV coordinated the survey work in Cuba, and CF coordinated the international 10/66 Dementia research Group program. MJP, CF and JRM assisted in the training of the Cuban investigators. MJP is the director of the 10/66 Dementia Research Group Program. MJP and JLB carried out the statistical analyses, and wrote the first draft of the paper. All authors read and approved the final manuscript.

## Pre-publication history

The pre-publication history for this paper can be accessed here:

http://www.biomedcentral.com/1471-2350/12/43/prepub

## Supplementary Material

Additional file 1 **List of SNPs used to estimate individual admixture**. Full list of 60 SNPs used to estimate individual admixture, with rs number, chromosome and genetic location (in centimorgans) Click here for file
