# Supplementary material for: Interactions between genetic admixture, ethnic identity, APOE genotype and dementia prevalence in an admixed Cuban sample; a cross-sectional population survey and nested case-control study
Source: BMC Med Genet. 2011 Mar 24;12:43. doi: 10.1186/1471-2350-12-43 (PMC3079615; doi:10.1186/1471-2350-12-43)
Supplement: Additional file 1 — List of SNPs used to estimate individual admixture. Full list of 60 SNPs used to estimate individual admixture, with rs number, chromosome and genetic location (in centimorgans) [file 1471-2350-12-43-S1.DOC]

| SNP rs number | Chromosome location | Genetic location (centimorgans) |
| --- | --- | --- |
| 1008984 | 1 | 212.712200 |
| 10752631 | 1 | 166.050290 |
| 1506069 | 1 | 245.855636 |
| 1780349 | 1 | 173.884583 |
| 2752 | 1 | 265.119366 |
| 2806424 | 1 | 171.312282 |
| 2814778 | 1 | 164.822103 |
| 6003 | 1 | 211.973061 |
| 723822 | 1 | 175.756640 |
| 905595 | 1 | 169.753084 |
| 1435090 | 2 | 76.029755 |
| 1861498 | 2 | 18.206342 |
| 3287 | 2 | 79.313151 |
| 1316579 | 3 | 193.884088 |
| 1465648 | 3 | 119.669717 |
| 17203 | 3 | 106.522997 |
| 768324 | 3 | 118.352080 |
| 938431 | 3 | 130.644659 |
| 1112828 | 4 | 149.028080 |
| 1403454 | 4 | 163.491982 |
| 719776 | 4 | 55.622617 |
| 1461227 | 5 | 170.585241 |
| 3309 | 5 | 70.454438 |
| 3340 | 5 | 171.275125 |
| 1935946 | 6 | 137.226972 |
| 2077681 | 6 | 7.422715 |
| 1320892 | 7 | 164.596490 |
| 2161 | 7 | 110.075478 |
| 2341823 | 7 | 148.328642 |
| 2396676 | 7 | 132.443180 |
| 1373302 | 8 | 101.784532 |
| 1808089 | 8 | 106.156739 |
| 1987956 | 8 | 134.364124 |
| 1327805 | 9 | 157.977084 |
| 1980888 | 9 | 113.037071 |
| 1594335 | 10 | 97.015769 |
| 1891760 | 10 | 140.112143 |
| 2207782 | 10 | 112.531122 |
| 1042602 | 11 | 112.302187 |
| 1487214 | 11 | 35.986372 |
| 594689 | 11 | 86.530676 |
| 5443 | 12 | 18.138176 |
| 726391 | 12 | 36.740486 |
| 2078588 | 13 | 74.381570 |
| 1153849 | 15 | 40.452622 |
| 2351254 | 15 | 97.078527 |
| 4646 | 15 | 46.480188 |
| 764679 | 16 | 26.003869 |
| 1074075 | 17 | 93.954865 |
| 2816 | 17 | 19.186852 |
| 717962 | 17 | 104.639257 |
| 1369290 | 18 | 106.372948 |
| 386569 | 19 | 81.461863 |
| 11467165 | 20 | 54.781902 |
| 718092 | 20 | 59.657466 |
| 16383 | 22 | 54.025776 |
| 878825 | 22 | 16.977601 |
| 1415878 | X | 143.825824 |
| 1986586 | X | 122.581488 |
| 2188457 | X | 141.823404 |

Additional File 1

List of 60 SNPs used to estimate individual admixture, with rs number, chromosome and genetic location (in centimorgans)
